# Effect of community-based intervention on improving access to treatment for sick under-five children in hard-to-reach communities in Niger State, Nigeria

**DOI:** 10.7189/jogh.09.010803

**Published:** 2019-06

**Authors:** Olusola Oresanya, Helen Counihan, Ibrahim Nndaliman, Ayodele Alegbeleye, Jonathan Jiya, Olatunde Adesoro, John Dada, Patrick Gimba, Lynda Ozor, Debra Prosnitz, Kolawole Maxwell

**Affiliations:** 1 Malaria Consortium, Abuja, Nigeria; 2 Malaria Consortium, London, UK; 3 Malaria Consortium, Niger, Nigeria; 4 Malaria Consortium (retired), Niger, Nigeria; 5 Niger State Ministry of Health, Niger, Nigeria; 6 World Health Organization, Abuja, Nigeria; 7 ICF, Fairfax, Virginia, USA

## Abstract

**Background:**

Access to prompt and appropriate treatment is key to survival for children with malaria, pneumonia and diarrhoea. Community-based services are vital to extending care to remote populations. Malaria Consortium supported Niger state Ministry of Health, Nigeria, to introduce and implement an integrated community case management (iCCM) programme for four years in six local government areas (LGAs). The objective was to increase coverage of effective treatment for malaria, pneumonia and diarrhoea among children aged 2-59 months.

**Methods:**

The programme involved training, equipping, ongoing support and supervision of 1320 community volunteers (CORPs) to provide iCCM services to their communities in all six LGAs. Demand creation activities were also conducted; these included community dialogues, household mobilization, sensitization and mass media campaigns targeted at programme communities. To assess the level of changes in care seeking and treatment, baseline and endline household surveys were conducted in 2014 and 2017 respectively. For both surveys, a 30×30 multi-stage cluster sampling method was used, the sampling frame being RAcE programme communities.

**Results:**

Care-seeking from an appropriate provider increased overall and for each iCCM illness from 78% to 94% for children presenting with fever (*P* < 0.01), from 72% to 91% for diarrhoea cases (*P* < 0.01), and from 76% to 89% for cases of cough with difficult or fast breathing (*P* < 0.05). For diagnosis and treatment, the coverage of fevers tested for malaria increased from 34% to 77% (*P* < 0.001) and ACT treatments from 57% to 73% (<0.005); 56% of cases of cough or fast breathing who sought care from a CORP, had their respiratory rate counted and 61% with cough or fast breathing received amoxicillin. At endline caregivers sought care from CORPs in their communities for most cases of childhood illnesses (84%) compared to other providers at hospitals (1%) or health centres (9%).This aligns with caregivers’ belief that CORPs are trusted providers (94%) who provide quality services (96%).

**Conclusion:**

Implementation of iCCM with focused demand creation activities can improve access to quality lifesaving interventions from frontline community providers in Nigeria. This can contribute towards achieving SDGs if iCCM is scaled up to hard-to-reach areas of all states in the country.

Pneumonia, diarrhoea and malaria are major causes of mortality in children under five years (U5) in sub-Saharan Africa. While pneumonia and diarrhoea account for 16% and 8% of deaths in U5 children respectively, this age group accounts for an estimated 66% of all malaria deaths, [1] potentiated by underlying malnutrition. These deaths can be prevented with proven cost-effective interventions, [2] which are often not available for children living in rural and remote communities located far from health facilities. This increases the risk of rapid progression to fatality due to delayed treatment [3]. In Nigeria, low coverage of life-saving high-impact preventive and curative childhood interventions is a key factor in high U5 mortality. In 2008, only 37% of children with diarrhoea received any rehydration or increased fluids to drink, with just 0.7% receiving zinc supplements, while the proportion of children with acute respiratory infections who were given antibiotics was 22.5% and of children with fever, only 6% received artemisinin-based combination therapy (ACT) [4]. Access to prompt and appropriate treatment are key to survival of children affected by these killer diseases [5,6].

Achieving universal health coverage is at the heart of the SDGs, [7,8] however, by definition this cannot be achieved without universal access [9]. Universal health coverage is said to be attained when people obtain the health services they need and are also protected from financial hardship due to out-of-pocket expenses and access to these services, meaning the opportunity or ability to do both [9,10]. The dimensions of access have been described as physical accessibility, financial affordability and acceptability in terms of willingness to seek services and not merely adequacy of supply [10,11]. Although many countries are far from universal coverage, they can take steps towards it by improving equitable access [12].

The United Nations Children’s Fund (UNICEF) and World Health Organization (WHO) released a joint statement on integrated community case management (iCCM) of childhood illnesses in 2012 [13]. Following this, Nigeria, adopted iCCM as part of its Child Health policy and developed national guidelines for its implementation in 2013.

iCCM is a proven equity-focused intervention for extending affordable care to hard to reach communities to reduce deaths among U5s [14]. Prompt and effective community management of pneumonia, malaria, and diarrhoea, has been found to reduce mortality by 70%, 60% and 70%-90% respectively [14,15]. It can address the three dimensions of universal access by bringing treatment services for children U5 closer to the home thereby eliminating the geographical or physical barrier to access; mitigating financial barriers to demand for services when provided free of charge [16] and promoting acceptability when delivered by trusted members of the community nominated by the communities themselves [17].

The flagship iCCM programme for Nigeria, Rapid Access Expansion (RAcE) programme iCCM programme for Nigeria launched in 2013 in Abia and Niger states was supported by WHO with funding from the Global Affairs Canada. Malaria Consortium supported Niger State Ministry of Health to introduce and implement iCCM for four years in six local government areas (LGAs). The objective was to increase the number of children 2-59 months receiving effective treatment for common illnesses through provision of iCCM services combined with demand creation activities for caregivers of young children. Children under five living in communities five kilometres or more from the nearest functional health facility were targeted.

Although a number of studies have documented the effect of iCCM on access to care in rural communities in African countries, [18,19] the acceptability and utilization of iCCM services provided by community volunteers in the Nigerian setting is yet to be documented. This study presents the findings from the endline assessment of the RAcE iCCM in Niger state, Nigeria.

## METHODS

### Programme implementation design

Niger State is located in North Central Nigeria and has a projected (2017) population of about 5 586 003. The state has the largest land mass in the country (76 263 km^2^) and majority of the population are spread across rural areas, with 30 percent of the population living in urban areas. Their occupation is largely agrarian and they are Muslim and Christian with a small minority practising traditional beliefs. Literacy rate is less than 50 percent and under-five mortality rate is 100 per 1000 live births. According to NDHS 2013, health seeking for children with fever, diarrhoea and acute respiratory infections is 38 percent, 42 percent and 29 percent respectively. Prevalence of global acute malnutrition is 6.1 percent, severe acute malnutrition is 0.5 percent while moderate acute malnutrition prevalence is 5.6 percent.

The Niger State RAcE programme was designed to increase the coverage of diagnostic, treatment and referral services through capacity building and operational support to health workers, communities and ministries of health at state and national levels. Over 161 973 children U5 in hard-to-reach areas of selected six LGAs were targeted to be reached through trained and equipped volunteer community caregivers known as community-oriented resource persons (CORPs). 1320 CORPs were trained and equipped to diagnose and treat children free of charge. The trained CORPs were provided respiratory timers, malaria rapid diagnostic test kits, amoxicillin dispersible tablets, ACT, ORS and zinc, as well as reporting tools including Sick Child Recording Forms and CORP Registers. Each CORP had on an average 118 children in his/her catchment population. The CORPs were supervised and mentored by community health extension workers (CHEWs) based at the primary health care centres, who were also trained on integrated management of childhood illnesses (IMCI). This system supported CORPs’ case management skills, as well as data collection and reporting needs. The supervision system followed the national recommendation of monthly visits to the CORPs by the CHEWs to check on competencies, supplies and record-keeping using standard checklists.

Eligible communities for iCCM were identified through a health facility assessment to ascertain functionality, and mapping of communities that were more than 5 km away from the nearest functional health facility. Selection of the CORPs was community-led and based on criteria recommended in the national guidelines, including residence in the community and ability to read and write. Under the RAcE Programme, CORPs and CHEWs participated in 6 days of iCCM training; the CHEWs had an additional three days training on supervision, which enhanced their capacity to supervise the CORPs. CORPs received an incentive of approximately 20 US dollars monthly to support transport costs for home visits and facility visits for replenishment of medicines and mentoring.

The behaviour change communication strategy designed and implemented for the programme, guided the development of appropriate messages and materials as well as innovative multi-channels to reach the caregivers and other key audiences. Community mobilization activities including community dialogues, household sensitization by specially trained social mobilisers, and mass media campaigns targeted at communities, were embarked on with the support of women’s, religious and community leader groups, to increase care-seeking and uptake of services as well as to augment CORPs’ credibility.

ICF International (ICF) provided technical support to strengthen routine data quality and programme evaluation surveys.

### Study design and objective

There were two cross-sectional household surveys conducted, one at programme baseline in 2014 prior to iCCM implementation, and the other at endline in February, 2017.

The objectives of the surveys were to assess care-seeking behaviour for sick children; coverage of iCCM assessment and treatment; and caregiver knowledge, attitudes, and practices related to pneumonia, diarrhoea, and malaria. Baseline and endline results were compared to assess changes in sick child care-seeking, assessment, and treatment coverage as well as caregivers’ knowledge of childhood illnesses and perceptions of CORPs’ services.

### Study setting

**Figure 1:**
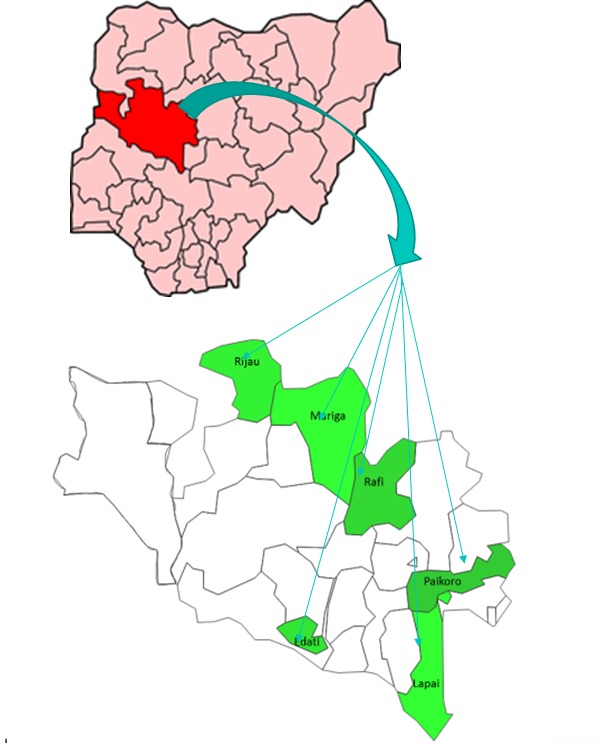
Study sites in Niger State, Nigeria.

Baseline and endline surveys were conducted across the six programme LGAs, Lapai, Paikoro, Rijau, Edati, Mariga and Rafi, before and after the intervention in 2014 and 2017 respectively (see **Figure 1**). There were no other community health interventions taking place in these LGAs during the programme implementation.

### Sampling and study size

Primary caregivers of children aged 2–59 months living in the programme communities who reported that their children had diarrhoea, fever, or cough with rapid breathing in the two weeks prior to the survey, were considered eligible for inclusion in the survey. A 30x30 multi-stage cluster sampling method [20] was used to select 30 clusters using probability proportional to size, the sampling frame being RAcE programme communities. In order to detect a 20 percent difference in the sick child indicators that include all sick children for a specific illness (fever, diarrhoea, or cough with difficult or fast breathing) between baseline and endline at 90 percent power with a two-tailed test and 95 percent confidence using cluster sampling, 263 cases were needed for each illness, this was however rounded up to 300 cases per illness.

### Data collection

Data was collected on key indicators related to caregiver knowledge of CORPs, caregiver perceptions of CORPs and care-seeking, assessment, treatment, referral adherence, and follow-up of sick children. To train the survey team, comprising of data collectors, supervisors and monitors, a training of trainers was held in Abuja from January 24 to 27, 2017 of supervisors and monitors while a cascade followed in Niger for the data collectors subsequently. Data collection was done using a standard household questionnaire comprising seven modules: caregiver and household background information; caregivers’ knowledge of iCCM activities in their community; caregivers’ knowledge of childhood illness danger signs; household decision-making; and a module for each major childhood illness: fever, diarrhoea, and fast breathing. The same questionnaires, which were developed by ICF and drawn from the Knowledge, Practice, Coverage (KPC) Survey tool, [21] were used for both baseline and endline except for the addition of two new questions at endline on whether caregivers sought care for their sick child and whether they sought care from a CORP. Participation in the study was voluntary, each provided written informed consent prior to interview. At the household level, the enumerator first determined whether an eligible child lived there. If there was an eligible child in the household, the interviewer administered the questionnaire, including all applicable illness modules, to the caregiver of the eligible child. If more than one child was eligible, and they were sick with different illnesses, their caregiver was asked about each instance of illness. If there was more than one eligible child in the household for an illness, the interviewer randomly selected one of the eligible children and interviewed his or her caregiver. If multiple children in the same household were reported to have symptoms in the preceding two weeks but had different caregivers, interviewers could interview multiple caregivers, as long as not more than one child from each household was included for each iCCM condition.

Quality control procedures during field work included daily spot checks by supervisors, during which they observed at least one interview per enumerator per day and also reviewed all completed questionnaires they received. In addition, one monitor was assigned to each LGA to ensure team compliance with the survey protocol and to provide further logistical, material, and technical support to the supervisors.

### Data analysis

The baseline and endline CSPro data files were imported into Stata v14 (Stata Corp, College Station, TX, USA) and merged into one file for the analysis. Point estimates and 95 percent confidence intervals were calculated for the survey indicators accounting for cluster effects. Pearson χ^2^ test was used to determine statistical significance for binary and categorical variables and regression for continuous variables. Changes between baseline and endline with p-values less than 0.05 were taken as statistically significant.

## RESULTS

### Participants background characteristics

A total of 899 primary caregivers of sick children were included in the survey at baseline and 630 at endline. **Table 1** shows the distribution of the disease condition among the eligible children surveyed. There was no significant difference in the sex and age distribution of the children, response rates for fever, diarrhoea, and cough with difficult or fast breathing cases targeted was 100 percent. Relatively more cases of fever, diarrhoea, and cough with difficult or fast breathing were captured at baseline (1,130) than endline (902) (**Table 1**).

**Table 1:** Characteristics of sick children included in the survey

Child characteristic	Baseline % (95% CI)	Endline % (95% CI)
**Sex of sick children included in survey:**		
Male, %	50.5 (47.0-54.0)	49.9 (45.0-54.7)
Female, %	49.5 (46.0-53.0)	50.2 (45.3-55.0)
**Age (months) of sick children included in survey:**		
2–11, %	20.2 (17.5-23.3)	15.0 (12.2-18.4)
12–23, %	21.1 (18.7-23.9)	22.1 (19.7-24.6)
24–35, %	17.1 (14.3-20.4)	22.1 (19.7- 4.6)
36–47, %	17.1 (14.3-20.4)	20.0 (17.1-23.3)
48–59, %	19.7 (16.8-22.9)	19.4 (16.5-22.7)
**Two-week history of illness of sick children included in survey:**		
Had fever, %	58.3 (52.9-63.5)	57.9 (53.9-61.9)
Had diarrhoea, %	47.9 (47.0-57.1)	46.9 (43.1-50.8)
Had cough with fast breathing, %	43.2 (38.1-48.4)	51.2 (47.7-54.7)
Average number of illnesses, N	1.5	1.6
**Total number of sick children included in survey:**	**899**	**680**
**Cases of illness included in survey:**		
Fever, N	413	301
Diarrhoea, N	374	300
Cough with fast breathing, N	343	301
**Total number of illness cases among sick children included in survey**	**1130**	**902**

CI – confidence interval

### Caregiver knowledge of illnesses and perception of CORPs

Over the course of program implementation, caregiver knowledge of two or more signs of childhood illness (eg, danger signs for sick children as well as symptoms and treatment of malaria and diarrhoea) increased significantly, from 56 percent at baseline to 68 percent at endline (*P* < 0.05) (see **Table 2**). Knowledge of the cause of malaria increased significantly, from 61 percent at baseline to 78 percent at endline, but knowledge of fever as a sign of malaria did not change significantly. Caregiver knowledge of correct malaria treatment (artemisinin-based combination therapy [ACT]) increased significantly between baseline and endline, from 26 percent at baseline to 73 percent at endline (*P* < 0.0001). Caregiver knowledge of correct diarrhoea treatment (oral rehydration solution [ORS] and zinc) also increased significantly, from 5 percent at baseline to 54 percent at endline (*P* < 0.0001).

**Table 2:** Caregiver knowledge of childhood illnesses

Caregiver illness knowledge	Baseline	Endline	*P*-value
**% (95% CI)**	**% (95% CI)**
Knows 2+ child illness signs	55.9 (45.9-65.4)	68.2 (58.9-76.3)	0.0463
Knows cause of malaria	61.2 (52.5-69.2)	77.8 (68.8-84.8)	0.0154
Knows fever is a sign of malaria	57.7 (49.8-65.2)	63.9 (54.9-72.1)	0.3194
Knows malaria treatment	25.5 (20.3-31.5)	72.9 (63.7-80.6)	<0.0001
Knows diarrhoea treatment (ORS + zinc)	4.9 (2.8-8.4)	53.5 (42.2-64.5)	<0.0001
**Total number of caregivers**	**721**	**510**	

CI – confidence interval

At endline, caregivers generally had positive perceptions of CORPs. Ninety four percent of caregivers viewed CORPs as trusted health care providers, and 96 percent believed that CORPs provided quality services, and were a convenient source of treatment, and the same percentage found the CORP at the first care-seeking visit (**Table 3**).

**Table 3:** Caregiver perceptions of iCCM-trained CORP

Caregiver perception of CORP	Endline	Endline N
**% (95% CI)**
Views the CORP as a trusted health care provider	94.1 (88.1-97.2)	474
Believes the CORP provides quality services	95.6 (89.7-98.2)	474
Cites the CORP as a convenient source of treatment	96.0 (90.7-98.3)	474
Found the CORP at first visit (for all instances of care-seeking included in survey)*	96.1 (93.7-97.6)	410

CI – confidence interval

*Includes only caregivers who report seeking care from a CORP for at least one sick child.

### Care seeking

**Figure 2:**
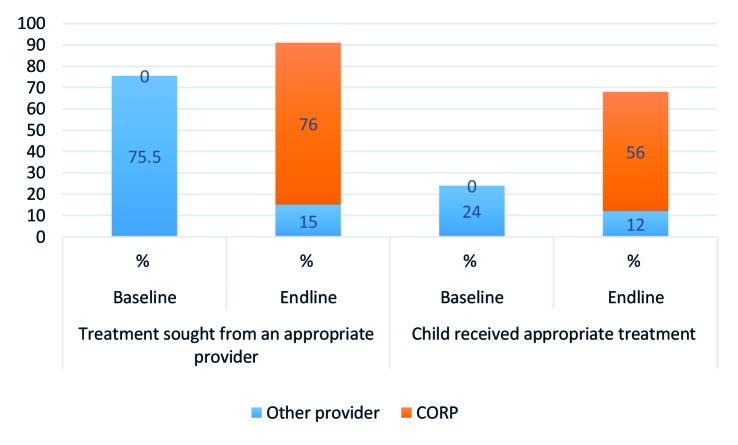
Contribution of CORPs to appropriate treatment.

**Table 4** presents results for all appropriate providers while **Figure 2** shows results for choice of CORPs as first source of care among those who sought any care for their children by illness. Overall, care-seeking for a sick child from an appropriate provider increased significantly, from 76% at baseline to 91% at endline *(P <* .001) as shown in **Table 4**. Care-seeking from an appropriate provider also increased for each iCCM illness from 78% to 94% for fever cases (*P* < 0.01), from 72% to 91% for diarrhoea cases (*P* < 0.01), and from 76% to 89% for cases of cough with difficult or fast breathing (*P* < 0.05).

**Table 4:** Care seeking from appropriate provider

Illness	Sought care from appropriate provider*		*P*-value	CORP was first source of care	Baseline N		Endline N
**Baseline % (95% CI)**	**Endline % (95% CI)**	**Endline % (95% CI)**			
Overall	75.5 (68.3-81.5)	91.4 (87.2-94.3)	0.0000	76.6 (65.9-84.8)	1130	902	
Fever	78.0 (70.6-83.9)	94.0 (88.8-96.9)	0.0001	78.4 (66.3-87.0)	413	301	
Diarrhea	71.9 (64.3-78.5)	91.3 (86.7-94.4)	0.0000	75.7 (64.8-84.0)	374	300	
Cough with fast or difficult breathing	76.4 (68.1-83.1)	88.7 (82.3-93.0)	0.0110	75.8 (64.4-84.4)	343	301	

CI – confidence interval

*Appropriate providers include hospital, health centre, health post, clinic, CORP, or pharmacy.

The results also showed that CORPs contributed largely to the increase seen at endline in the percentage of children who received treatment from an appropriate provider. **Figure 2** shows a significant increase in the proportion of children who received care from an appropriate provider from 75 percent at baseline to 91 percent at endline, with CORPs contributing 76 percent and other providers 15 percent. Of those who sought care from appropriate providers at baseline, only 24 percent received appropriate treatment compared to 68 percent at endline, out of which CORPs constituted 56 percent.

**Figure 3:**
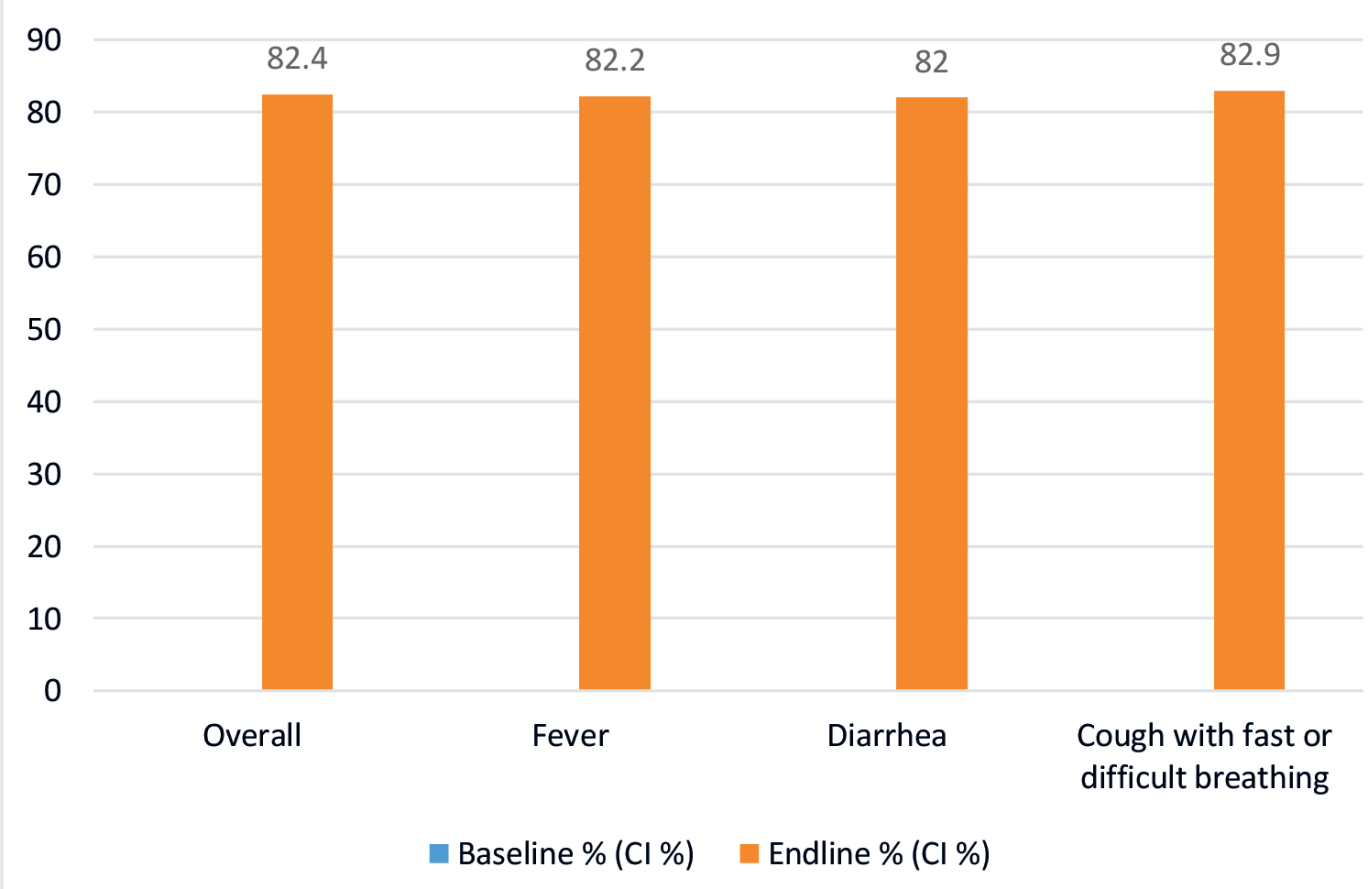
Care seeking from CORPs as first source of care by illness.

In addition, source of care-seeking practices shifted substantially, with caregivers at endline selecting to seek care from CORPs in their communities for the majority of cases of all illnesses experienced by their children. Among those who sought any care, more than 82 percent of fever, diarrhoea and cough with fast or difficult breathing cases sought care form CORPs as the first source of care at endline. See **Figure 3**.

**Figure 4:**
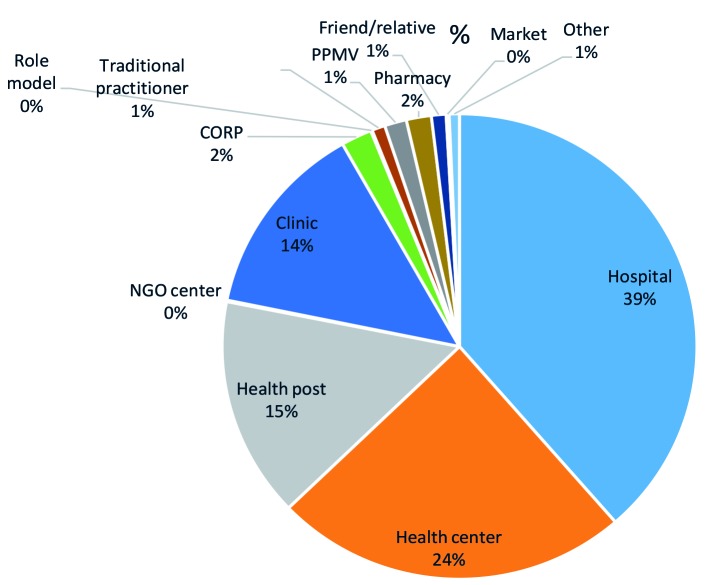
Percentage of caregivers who sought advice or treatment for his or her sick child from a given location as the first source, among those who sought any care at baseline.

**Figure 5:**
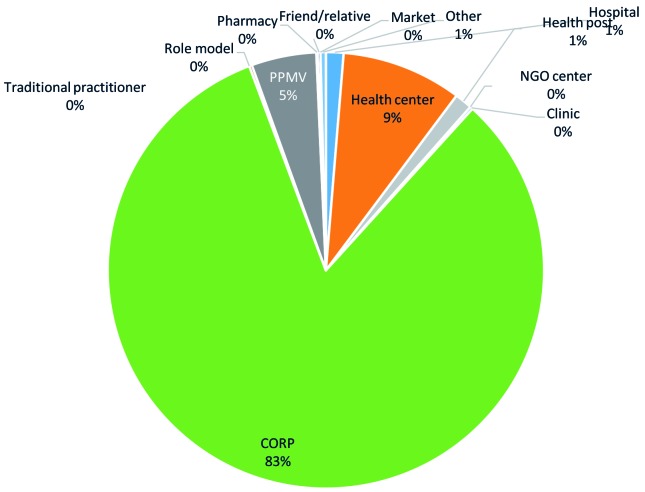
Percentage of caregivers who sought advice or treatment for his or her sick child from a given location as the first source, among those who sought any care at endline.

When analysed by type of facilities visited, a shift was noticed from hospitals and other health facilities as first source of care to CORPs. The baseline and endline pie charts in **Figures 4** and **Figure 5** show that among all cases of illness for which care was sought at baseline, when there were no CORPs, the majority of caregivers sought care at a hospital (39 percent) or a health centre (24 percent); by endline, the majority of cases of illness sought care from CORPs (83 percent), followed by health centres (9 percent) with a significant drop in the cases that sought care at hospital as first source of care to one percent.

### Sick child assessment and treatment

Overall, appropriate diagnosis and treatment of fever, diarrhoea and cough with fast or difficult breathing increased at endline. Among cases of fever who sought care from a CORP at endline, 77 percent received a malaria test, 90 percent of the caregivers reported receiving the result of the test, and 89 percent reported receiving ACT after a positive test (**Table 5**). Among children with fever who sought care from a provider other than a CORP at endline, 57 percent received a malaria test from the provider, 41 percent of their caregivers reported receiving the result of the test from the provider, and 48 percent received ACT from the provider after being confirmed for malaria. These results indicate overall more appropriate assessment and treatment of fever cases by CORPs, compared to cases seen by other providers. The findings were similar for assessment of children with cough with fast or difficult breathing; overall, the proportion of children who had cough with fast or difficult breathing that had their respiratory rates assessed increased significantly from 48 percent at baseline to 62 percent at endline.

**Table 5:** Malaria and fast breathing assessment among children with fever or cough*

Malaria and fast breathing assessment	Baseline	Endline	*P*-value	Baseline N		Endline N
**% (95% CI)**	**% (95% CI)**
**Fever cases in which care was sought from CORP:**						
Child had blood drawn by CORP	0	77.1 (67.6-84.4)	0.0003	17		240
Caregiver received result of malaria test	0*	90.3 (0.8-1.0)	na	0		185
Blood test positive for malaria	0*	92.8 (0.9-1.0)	na	0		167
Received ACT after positive malaria test, among those who had a positive malaria test	0*	89.0 (0.8-1.0)	na	0		155
**Fever cases in which care was sought from providers other than CORP:**						
Child had blood drawn by other provider	99.4 (95.3-99.9)	57.1 (40.9-72.0)	0.0000	318		112
Caregiver received result of malaria test	27.9 (21.1-35.8)	40.6 (28.8-53.6)	0.0797	316		64
Blood test positive for malaria	79.6 (65.0-89.1)	96.2 (73.8-99.6)	0.0708	88		26
Received ACT after positive malaria test, among those who had a positive malaria test	68.6 (53.1-80.8)	48.0 (28.0-68.7)	0.0546	70		25
**All cough with fast or difficult breathing cases:**						
Respiratory rate assessed	47.8 (40.8-54.9)	62.1 (51.9-71.4)	0.0153		343	301
**Cough with fast or difficult breathing cases in which care was sought from CORP**						
Respiratory rate assessed	0†	55.8 (44.1-67.0)	na		17	231
**Cough with fast or difficult breathing cases in which care was sought from provider other than CORP:**						
Respiratory rate assessed	57.4 (50.0 – 64.5)	42.3 (30.9 – 54.3)	0.0161		256	102

n/a – not applicable, ACT – artemisinin-based combination therapy, CORP – community-orientated resource person

*Some cases sought care from multiple providers, which is why the sum of the endline N for those assessed by CORP and those assessed by other providers (333) is larger than endline N that had respiratory rate assessed (301) for fast breathing.

†There were no cases or the number of cases was too small to calculate a percentage.

Among confirmed malaria cases, the percentage of children who received ACT within the same or next day following the onset of fever increased significantly, from 57 percent at baseline to 74 percent at endline (*P* < 0.05) (see **Table 6**). For cases of diarrhoea, treatment with ORS and zinc by any provider increased significantly, from 13 percent at baseline to 74 percent at endline (*P* < 0.001), and for cases of cough with difficult or fast breathing, treatment with amoxicillin by any provider increased significantly from 29 percent at baseline to 61 percent at endline (*P* < 0.001). At endline, treatments received from CORPs contributed significantly to the overall increase in prompt and appropriate treatments received by children 2-59 months compared to other providers. 61 percent out of 74 percent treated with ACTs following confirmation of malaria, were treated by CORPs. CORPs also contributed 75 percent to ORS treatment, 68 percent to zinc and 66 percent to ORS and zinc and 47 percent to amoxicillin treatment for fast breathing at endline.

**Table 6:** Appropriate treatment coverage

Illness (treatment)	Baseline	Endline	*P*-value	Baseline N	Endline N
**% (CI %)**	**% (CI %)**
**Received treatment:**					
Confirmed malaria (ACT same or next day following the onset of fever)*	56.9 (46.5-66.8)	73.9 (64.7-81.4)	0.0123	72	188
Diarrhoea (ORS)	68.2 (59.5-75.7)	88.3 (81.9-92.7)	<0.0001	374	300
Diarrhoea (zinc)	15.0 (10.5-20.9)	77.0 (66.8-84.8)	<0.0001	374	300
Diarrhoea (ORS and zinc)	12.8 (8.9-18.2)	74.0 (63.6-82.3)	<0.0001	374	300
Cough with difficult or fast breathing (amoxicillin)	28.6 (21.6-36.7)	60.5 (50.2-69.9)	<0.0001	343	301
**Received treatment from CORP:**					
Fever (ACT same or next day following the onset of fever)	0	55.5 (46.6-64.0)	na	413	301
Confirmed malaria (ACT same or next day following the onset of fever)*	0	60.6 (50.0-70.4)	na	72	188
Diarrhoea (ORS)	0	75.3 (63.9-84.0)	na	374	300
Diarrhoea (zinc)	0	68.3 (57.5-77.5)	na	374	300
Diarrhoea (ORS and zinc)	0	66.3 (55.5-75.7)	na	374	300
Cough with difficult or fast breathing (amoxicillin)	0	47.2 (36.3-58.3)	na	343	301
**Received treatment from provider other than CORP:**					
Fever (ACT same or next day following the onset of fever)	25.2 (20.0-31.2)	9.0 (4.5-17.1)	0.0015	413	301
Confirmed malaria (ACT same or next day following the onset of fever)*	56.9 (46.5-66.8)	13.3 (7.1-23.4)	<0.0001	72	188
Diarrhoea (ORS)	65.0 (55.5-73.4)	13.0 (7.6-21.4)	<0.0001	374	300
Diarrhoea (zinc)	12.8 (9.0-18.0)	9.7 (6.4-14.3)	0.3157	374	300
Diarrhoea (ORS and zinc)	11.8 (8.1-16.8)	7.7 (4.5-12.7)	0.1541	374	300
Cough with difficult or fast breathing (amoxicillin)	24.8 (18.0-33.1)	13.3 (8.1-21.0)	0.0212	343	301

CI – confidence interval, ACT – artemisinin-based combination therapy, na – not applicable, CORP – community-orientated resource person

*Denominator for confirmed malaria is restricted to those with a positive malaria diagnostic test result.

## DISCUSSION

Over the course of the intervention, there was significant increase in caregiver knowledge of two or more danger signs of childhood illness, cause of malaria, correct malaria treatment and correct diarrhoea treatment. Most caregivers of sick children, especially those who live in rural and remote communities, do not seek care from trained health personnel, [22] partly due to the knowledge level of caregivers. Studies have documented the relationship between knowledge of caregivers and care seeking [23-25]. Bruce et al in a study of the determinants of care seeking for children with pneumonia and diarrhoea in Guatemala documented mother's perception of illness severity and recognition of WHO-defined danger signs as predictors of care seeking for childhood illnesses [26]. Another study in Pakistan concluded that community mobilization and behaviour change activities should be included in community case management programmes rather than isolated strengthening of skills of community health workers (CHWs) [27]. The increase found in Niger state was likely boosted by the demand creation activities and behavioural change communication strategy implemented as part of the iCCM programme. Caregivers were targeted as the primary audience with the aim of improving their knowledge on childhood illnesses and promoting uptake of services provided by CORPs.

Caregivers in Niger State had positive perceptions about CORPs as trusted health care providers who were a convenient source of treatment and provided quality services. It is generally agreed that CORPs as community volunteers are critical in improving access to life saving interventions, however it is important that they are accepted and trusted by the communities they serve. Poor confidentiality and trust have been identified as key barriers to CHW acceptability in delivering maternal and child health services in the home [28,29].

Overall, this study found care-seeking for a sick child from an appropriate provider increased significantly for all three diseases of focus with a shift towards the CORPs in the communities as the first source compared to other sources of care. This fits with similar findings from previous studies which suggests that iCCM influences local care-seeking practices and reduces workload at already over-burdened health facilities [28,30,31]. We are not aware of any other programme being implemented during the project period which may have also contributed to the outcomes on health seeking behaviour presented here.

The study demonstrated a significant increase in diagnosis and treatment coverage for malaria, cough with fast or difficult breathing and diarrhoea in a timely manner with major contributions by CORPs as appropriate providers. Although interventions promoting care seeking improve mortality outcomes, timely care seeking from an appropriate care provider is also crucial [32]. Timely treatment is especially vital for children with symptoms of malaria and pneumonia, as improved outcomes have been associated with provision of treatment within 24 hours of onset of symptom [33]. The increased coverage could be attributable to the fact that 96 percent of the caregivers in the study perceived CORPs as a convenient source of care closer to the home and that they also provided quality services. Studies have documented a number of factors that influence care-seeking behaviour, which include perceptions of cause of illness, distance, cost, socio-cultural barriers, knowledge and information barriers and quality of available care among others [34,35]. The increased care seeking and treatment coverage found in this study further re-affirm the documented ability of iCCM to address geographical or physical barriers and improve access to care as evidenced by increased coverage of treatments [19,36-38].

Overall, there was better assessment and appropriate treatment of fever and suspected pneumonia cases and by CORPs, compared to cases managed by other providers, implying that community volunteers when trained, supported and well equipped can implement iCCM according to national guidelines. In all intervention areas, children with fever taken first to CORPs were significantly more likely to receive a test, to receive the test results, and to receive treatment with ACT than children taken to other providers, including primary health centres (PHCs). Children seen by CORPs were also more likely to have their respiratory rates assessed. This finding is comparable to a Mozambique study which found that only 41 percent of children with fever treated by first level health facility workers were tested with an RDT, and 19 percent of them received respiratory rates assessment compared to 60 percent and 68 percent of children seen by CHWs respectively [39]. It is however important to note that even though CORPs performed better than other providers in child assessment, the proportion of children who had their respiratory rates counted in this study remained low (56 percent). We also found that CORPs were less likely to treat children with fast breathing with amoxicillin than they were to treat cases of malaria and diarrhoea. Although reasons for these differences were not measured in this survey, it calls for closer supervision and mentoring of these community volunteers to ensure they are adhering to protocol.

A number of factors could have contributed to the sub-optimal performance of other providers, including PHCs, found in this study. Lack or outdated knowledge of treatment protocol or algorithm, non-availability of commodities for testing and treating the child, as well as lack of motivation and poor supervision, are possible factors as documented in other studies [40-42]. In order to maintain quality in the continuum of care, which should not be neglected for the sake of universal access, primary health care facilities, which should play supervisory roles for CORPs, must also be strengthened in terms of adherence to protocol, supportive supervision and uninterrupted supplies [43]. The overall benefit of iCCM in achieving universal access can only be harnessed when scaled up with good quality of care; and this will require not only community ownership of the intervention but also political commitment of governments to invest in it as required elements of sustainability.

### Limitations

A major limitation that should be recognized while interpreting the results of this study is the that results of treatment of confirmed malaria are based on caregiver recall of receiving a finger or heel stick (rapid diagnostic test [RDT] or microscopy, depending on the provider), which can be poor [44].

## CONCLUSION

Implementation of iCCM with focused demand creation activities contributed to improved timely and appropriate treatment for fever, diarrhoea and cough with fast or difficult breathing for children living in rural and remote areas in Nigeria. Scaling up of iCCM, using frontline community providers, to hard-to-reach areas across Nigeria can contribute towards achieving the SDGs.
